# Probing dynamic myocardial microstructure with cardiac magnetic resonance diffusion tensor imaging

**DOI:** 10.1186/s12968-014-0089-6

**Published:** 2014-11-12

**Authors:** Leon Axel, Van J Wedeen, Daniel B Ennis

**Affiliations:** Departments of Radiology and Medicine, NYU School of Medicine, New York, NY USA; Department of Radiology, Massachusetts General Hospital, Harvard University School of Medicine, Boston, MA USA; Department of Radiological Sciences, University of California, Los Angeles, CA USA

**Keywords:** Cardiovascular magnetic resonance, Diffusion tensor imaging, Heart, Sheetlets, Strain, Hypertrophic cardiomyopathy

## Abstract

This article is an invited editorial comment on the paper entitled “In vivo cardiovascular magnetic resonance diffusion tensor imaging shows evidence of abnormal myocardial laminar orientations and mobility in hypertrophic cardiomyopathy” by Ferreira et al., and published as Journal of Cardiovascular Magnetic Resonance 2014; 16:87.

## Myocardial microstructure

**Figure 1:**
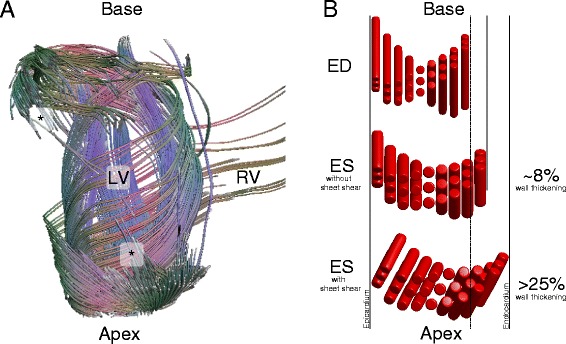
**Myocardial structure and function.** **(A)** Diffusion tensor “fiber” tracking of a canine heart reveals large-scale connectivity of the end-to-end anastomoses of continuously branching myocytes. With sufficiently high spatial resolution (especially required at the apex and base) the principal eigenvector can be tracked from asterisk (*) to asterisk (*) while tracing out aspects of the base, apex, endocardium, and epicardium. **(B)** A representative sheetlet structure comprised of three myocyte layers. Incompressible myocyte shortening of ~15% gives rise to only ~8% radial wall thickening when sheet-shear is absent. In the presence of sheet shear, which is accommodated by the sheetlets, radial wall thickening increases to >25%.

The microstructure of the heart is complex, but highly organized. The complexity of the heart’s organization was first quantitatively described in the well-known work of Streeter et al. [1,2], wherein it was shown that the ventricular heart wall is locally composed of coherently aligned myocytes, whose local average “fiber” orientation (helix angle) changes systematically from epicardium to endocardium. When viewing the epicardial surface of the heart, the sub-epicardial “fibers” are obliquely directed and smoothly transition to more hoop-like midwall fiber directions, and then to subendocardial fiber directions with an opposite obliquity. An example of such variation of the myocyte orientations across the heart wall is seen in Figure 1 of the article in the Journal of Cardiovascular Magnetic Resonance (JCMR) by Ferreira et al. under discussion here [3]. The orientation of the myocytes is conventionally measured with respect to a locally defined circumferential direction, with the epicardial myocytes having a negative helix angle and the endocardial myocytes having a positive helix angle. There is, in fact, a large-scale organization of myocytes that is observable from “fiber tracking” in ex vivo diffusion tensor MRI (DT-MRI) data (Figure 1A), which suggests that tracing along the direction of any one myocyte will tend to lead you back to the same myocyte – an idea that accords with the geometric requirement that a continuous vector field (considering the long axis of each myocyte as a vector, along which stress is exerted in contraction) can only topologically be accommodated on a toroid. In fact, the myocytes are reported to form a continuously branching functional syncytium, with splitting and merging of branches every few myocytes; thus we cannot strictly talk about distinct fibers, per se, in the myocardium, and we will herein avoid the terms “fiber” and “myofiber”, which are largely borrowed from the skeletal muscle literature.

Myocyte contraction during systole leads to the observed circumferential and longitudinal shortening of the ventricle and concomitant radial thickening, along with the twisting of the apex relative to the base that comprises a torsional component of the ventricular contraction. Interestingly, myocytes isovolumically shorten by ~15% during systole, which only accords with an ~8% increase in myocyte radius (Figure 1B). This is insufficient to account for the observed radial wall thickening, which is upwards of 25%. Hence, to generate >8% wall thickening another mechanism is needed. The work of Spotnitz et al. [4] was the first to highlight the fact that the countable number of myocytes across the ventricular wall increases from end diastole to end systole, at a given level! For this to be possible, the myocardium must undergo a large shear deformation that rearranges the relative position of the myocytes and accounts for the large amounts of wall thickening (Figure 1B). This hints at the idea that there must exist a structural element that facilitates large shear deformations (e.g. the sliding of myocytes over one another).

More recently, it has become clear that a secondary “sheet” structure is a fundamental constituent of the heart’s microstructure; this consists of a laminar arrangement of discrete structures a few myocardial cells in thickness and length, separated by cleft-like potential spaces [5]. While the end-to-end anastomoses of branching myocytes characterizes the longitudinal nature of the syncytium, the lateral branching of the sheets reflects the transverse syncytial nature underlying the structure of the myocardium. These “sheets” are locally oriented perpendicular to the local myocyte orientation, and they tend to fall into two dominant families, which are approximately perpendicular to each other [6,7]. Confocal micrographs of myocardial structure are particularly revealing [8,9]. It has been shown that shear between adjacent sheets contributes significantly to the radial wall thickening component of cardiac contraction [10,11]; it is likely that the sheets reduce the resistance to shearing within the heart wall during contraction, thus relieving some of the internal stresses that would otherwise build up during systole. The relative increase in systolic wall thickening associated with the changing angulation of the sheets is somewhat like the structure of a venetian blind increasing in apparent thickness while being opened. When a material releases internal stress via motion along a family of internal clefts, these clefts may be called a “slip system”. For maximum effectiveness, the slip planes will coincide with the directions of maximum shear, which occur at an angle of 45° to the direction of maximum strain. (Think of a deck of cards on the table: the slip system is horizontal while maximum elongation and contraction occur at + and – 45°.) In the myocardium, the direction of maximum strain is radial, and so the two populations of sheets may represent planes with one axis containing the myocardial fiber, and the other axis at angles of ±45° to the radial direction. In this issue of JCMR, Ferreira et al. use the term “sheetlet,” a term that has been used previously [12]; we find this to be a concise and accurate description of the sheet structure, which should be pictured as composed of discrete laminae of short extent (many myocytes), whereas the term “sheet” (or “myolaminae”) might be misinterpreted as representing very large scale structural features, which are not supported by the evidence to date. Thus, we will adopt the term “sheetlet” herein for these structures.

The mechanism by which sheetlets fail to align as macroscopic sheets is not widely known, but is quite interesting. In 3 dimensions, fields of planes seldom have 2D surfaces mutually tangent to any finite region of them: sheetlets, but no sheets. This is quite different from the 1D case. There, a continuous field of little lines in 2D or 3D is a vector field, and every continuous vector field is tangent to a family of continuous 1D curves. The undesirable nature of macroscopic sheets or cracks in the myocardium is clear, for they may allow it to disassemble like pages in a book or layers of an onion. However, the twisting character of the myocardial sheetlets may have a more positive function: while allowing transverse slip and shear, their mutual non-alignment serves to distribute mechanical stress in three dimensions. When we tear a piece of paper, for example, we rely on the fact that stress always concentrates and re-concentrates the propagating break point. The twisting sheets of the myocardium allow them to behave more like a rope, in which stresses rapidly redistribute laterally, fiber to fiber, so as to be borne equally over a much larger volume.

## Imaging myocardial microstructure

While these features of the microstructure of the heart wall are invisible with the usual macroscopic methods of imaging the heart, diffusion-weighted magnetic resonance imaging (DWI) of the heart [13] is a relatively new imaging method that renders the effects of microstructural features on the self-diffusion of water visible. In DWI, after MR excitation of the tissue water, a pair of pulsed magnetic field gradients along a chosen direction are used to first impose a very short wavelength periodic spatial variation (“winding”), along that direction, of the phase of the local signal-producing water molecules in the tissue and then (after a delay) to reverse it (“unwinding”), prior to the magnetic resonance signal detection [14]. If there is no net motion of the water molecules along the direction of the applied gradient between the pair of gradient pulses, the signal will be restored to the original strength that it would have had without the gradient pulses. However, if there is a sufficient amount of random motion (i.e. diffusion) of the water molecules away from their initial positions in between the two gradient pulses, the phase unwinding process will be incomplete, leading to phase dispersion, and the net observed signal will be correspondingly reduced.

The anisotropic tissue microstructure of the heart affects the relative amounts of diffusional motion along different directions; therefore, repeating the DWI process with diffusion-sensitizing gradients applied in different directions allows for characterization of spatially anisotropic diffusion. With a sufficient number of such measurements, the diffusion coefficient can be characterized by a six-component tensor; this is called diffusion tensor MRI (DTI). The six-component diffusion tensor can be used to calculate an equivalent set of three mutually perpendicular “eigenvectors,” with associated directions and magnitudes (eigenvalues), with the primary eigenvector corresponding the direction of the greatest diffusion (i.e. fiber direction) and the tertiary eigenvector corresponding to the direction of the least diffusion (i.e. sheet-normal direction). DTI has been most widely applied to in vivo imaging of the brain, to map the pathways of the nerve connections through their corresponding effects on the local directionality of the diffusion. It has also been applied to both ex vivo [15,16] and in vivo imaging of the heart [17]. However, in vivo DTI of the heart is very difficult, due to the technical challenge of probing for incoherent diffusional motion (on the order of microns) on a background of much larger coherent bulk wall motion (on the order of millimeters). Thus, most cardiac diffusion studies to date have focused on DTI of heart specimens, enabling production of elegant 3D descriptions of the myocyte and sheetlet structures of the ex vivo heart wall (often after many hours of image data acquisition) [18,19], while in vivo cardiac DTI studies have remained relatively limited.

Three principal approaches have been used to separate the effects of diffusional and bulk motion in in-vivo DTI: 1) Perform the winding and unwinding diffusion-sensitizing gradients at the same relative time in subsequent heart cycles (storing the local phase information in the longitudinal magnetization of a stimulated echo, so that it persists over a heart cycle) and the bulk motion is approximately compensated. This approach provides a relatively long time for diffusion effects to evolve, thereby improving detection, but it can also lead to large signal losses, due to subtle differences in bulk motion that may occur between heart beats [13]. 2) The winding and unwinding parts of the diffusion encoding process can be applied at matched “sweet spots” within a given cardiac cycle, so that the strain effect over the cardiac cycle is approximately balanced [20]. 3) The winding and unwinding parts of the diffusion encoding process can be applied with much shorter intervals between them, using conventional spin echo signals, at times within the cardiac cycle when the heart remains approximately stationary. This provides only a relatively short time for diffusion effects to evolve, but it can also lead to less bulk motion signal losses [21]. The advent of gradient hardware with very high maximum gradient amplitudes (80mT/m or higher) is making this last approach increasingly practical.

In analyzing the resulting DT-MRI signal data, we need to: 1) allow for the possible effects of other sources of signal loss (e.g. pseudorandom motion of the blood within the microcirculation [22], or phase dispersion due to shearing or rotation within the signal-producing voxel); and 2) consider the need for measurements along an adequate number of different diffusion-encoding directions in order to capture the anisotropic structure of the tissue [23] (while a simple diffusion direction distribution can be characterized by a symmetric tensor with six independent components, this may not be adequate to describe diffusion in more complex structures). We also must consider the potential need to account for the effects of tissue deformation (“strain”) during the diffusion measurement time on the apparent diffusion. Even with an isotropic diffusion coefficient, shortening of the tissue along one direction between the times of application of the diffusion-sensitizing gradient pulses would produce an apparently greater relative amount of diffusion (relative to the underlying tissue) along that direction, while an associated stretching along another direction would produce an apparent reduction of the relative diffusion along that direction. Similarly, in the case of shearing of sheetlets relative to each other, the associated bulk motion may lead to an apparent increase in diffusion for transverse motion of water molecules across the cleft between the sheetlets, with the changing contractile state of the heart.

When first described, the effect of myocardial deformation on the measured myocardial diffusion was considered a kind of artifact in the imaging, but on further consideration, this effect provides a window into a novel aspect of physiology. When myocardial diffusion is encoded over an entire cardiac cycle, one has observed a valid representation of the pattern and extent of random Brownian motion of water molecules over one heartbeat. As water moves, so move other small molecules in water solution. While the exact scale of motion will vary with molecular weight and other factors, the water diffusion is nonetheless representative of local physics and geometry common to all small molecules, including oxygen. It follows that if the water diffusion is observed to be different during material strain over a certain time period, then molecular transport will be affected similarly. Interestingly, this interaction between diffusion and strain can have an impact on the net transport. For this to happen, it is only necessary that the tensors of diffusion and of strain are not proportional, which they are not in the normal heart. For example, the direction of maximum shortening is not coaxial with the myocyte’s long axis at all wall depths [24]. Simple numerical calculations suggest this mechanism can augment molecular transport at least 15% in a continuum model and perhaps more than 30% with estimated effects of sheet slippage. In short, strain improves perfusion and exchange. Conversely, if a segment becomes stiff or hypokinetic, this advantage will disappear.

Hypertrophic cardiomyopathy (HCM) is characterized by regional (e.g. septal) or diffuse hypertrophy of the myocardium, which is not associated with increased pressure loading; it can be associated with a wide range of different underlying alterations in the contractile apparatus of the myocyte [25]. Histologically, in HCM there is a characteristic loss of the usual highly ordered local myocyte orientation seen in normal hearts [26]. There is also an increase in the amount of collagen in the heart wall, especially around blood vessels [26]. HCM patients may also develop local fibrosis within areas of hypertrophy, which can be seen as corresponding local areas of abnormal late gadolinium enhancement; these areas of fibrosis may be associated with an increased risk of arrhythmias [27]. Although global functional measures of the heart (such as the ejection fraction) may be preserved in HCM, there is typically a significant reduction in the degree of local contraction and rates of relaxation of the affected muscle [28,29], which may be related to the altered local tissue structure in HCM; there are also alterations of the diastolic function in HCM, which may be related to alterations in calcium handling [29]. Symptoms in HCM are primarily related either to mechanical obstruction of the left ventricular outflow tract, due to associated structural alterations, or to arrhythmias (which can lead to sudden death).

Currently, there are several open questions related to DTI of the heart, including: 1) How can we most reliably and efficiently apply DTI techniques to the heart in vivo? 2) What clinically useful information can we hope to extract from DTI, beyond simply demonstrating the wall’s depth-dependent myocyte orientation structure? 3) How can we relate observed changes in cardiac DTI-derived measures to corresponding alterations in the structure and function of the underlying myocardium? The work of Ferreira et al. helps us to begin answering these questions.

### The paper by Ferreira et al

**Figure 2:**
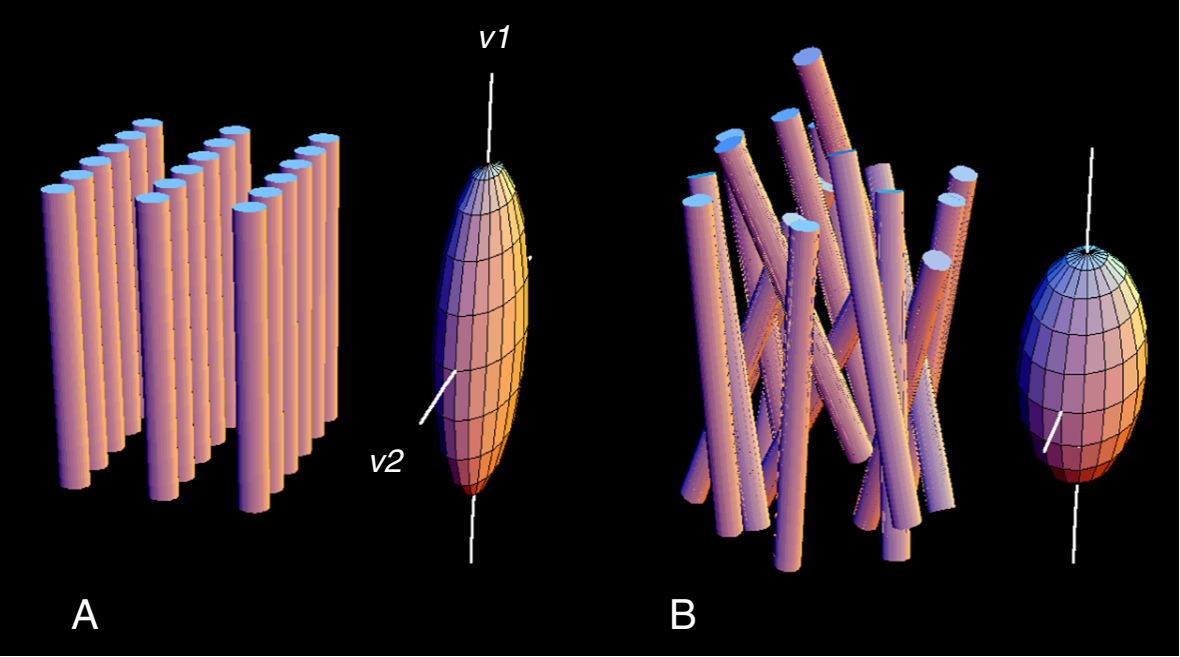
**Schematic representation of architecture of fibers and sheets of normal myocardium (A) and in myocardial fiber disarray (B), and of their respective diffusion tensors.** In the normal, fibers are locally parallel, and organized in local planes, sheetlets. Diffusion tensors represent an ellipsoid whose leading eigenvector (v1), the direction of maximum diffusion, is aligned with the mean axis of the myocardial fibers, and whose second eigenvector (v2) indicates the local sheet orientation. These directions are well-defined in the normal heart **(A)**, and their distinctiveness is reduced or lost in fiber disarray. This is reflected in the lengths of the eigenvectors, which become more nearly equal in disarray **(B)**. In maximum disorder, the diffusion tensor is a sphere, without distinguished directions. Details omitted include the facts that myocardial fibers are not isolated but branched syncytia, and that disarray is typically accompanied by increased matrix protein and non-contractile cells.

In JCMR, Ferreira et al. [3] report the use of sets of multiple DTI data acquisitions (using single-shot EPI readouts), that each span a whole cardiac cycle, but are centered on either mid-diastole or end-systole, in order to look for differences in the corresponding directions of the myocyte and sheetlet orientations across the cardiac cycle. This approach was applied to image the midlevel of the ventricles, in both control subjects and patients with HCM. No attempt was made to account for possible effects of the varying strain effects over the cardiac cycle on the apparent diffusion. Modeling the diffusion coefficient as a six-component tensor provided the directions of the primary (E1, assumed to correspond to the local myocyte direction) and secondary (E2, assumed to correspond to the principal local sheetlet direction) diffusion eigenvectors. These eigenvectors were used to calculate two corresponding angles in a cardiac-centered reference frame: the myocyte helix angle (HA) and the sheetlet angle (E2A). The expected depth-dependent variation in HA was found in both control and HCM subjects, without much difference between end-diastole and end-systole; in Streeter’s original report, there was seen to be a “relatively constant change in angle through the wall” in systole and diastole. It is perhaps somewhat surprising that there was no significant difference found between the observed control and HCM HA values, given the difference in degree of orientation of the myocytes in HCM that might be expected with the so-called “disarray” that is used to describe the appearance in HCM (although there is little data on the actual distribution of myocyte orientations in HCM). However, this similarity of the HA may just reflect a consistent net intravoxel average orientation, without reflecting a likely broader underlying distribution of intravoxel HA in HCM. This expected difference in the intravoxel HA distribution function may result in significant differences in the magnitudes of the associated eigenvalues (Figure 2), but this data is not reported in this paper. There were, however, significant cardiac cycle-dependent changes seen in E2A, with E2A becoming more perpendicular to the wall in measurements made at end-systole in control subjects, as might be expected for the sheetlet orientation changes that contribute to wall thickening. This change was much reduced in HCM patients, where the orientation of E2A remained relatively perpendicular to the wall in measurements made at both mid-diastole and end-systole, particularly in thickened regions.

In seeking to interpret these reported observations on differences in the apparent orientations of the sheetlets in measurements made at mid-diastole and end-systole, and differences in these changes between control and HCM subjects, we find some major difficulties and limitations: 1) It is somewhat unclear what the observed differences in the secondary diffusion eigenvector in measurements made at different cardiac cycle phases would actually represent, since the measurements that are made are dependent on the history of the diffusion orientations, and thus may also be affected by associated changes in the relative effective diffusion coefficients over the whole cardiac cycle. Thus, they may also be dependent on the varying strain history, as well as the local orientation at the time of the signal detection, as discussed further below. 2) The use of a single angle to represent the orientation of the sheetlets within a voxel is potentially misleading, as the heart wall is known to be populated with two different families with significantly different distributions of orientations in normal subjects [7] (and with unknown, but likely poorly aligned, orientation distributions in HCM). The histologic specimen images provided in the paper (their Figure eight D and E) apparently show just such a heterogeneous mixture of local sheet orientations, within a region smaller than an imaging voxel. 3) The lack of measurements of the associated strain changes within the myocardium (e.g. with magnetization tagged MRI) reduces the ability to assess the degree to which they may have contributed to the findings. However, even if these were available, the uncertainty about relevant aspects of the tissue microstructure, and about the nature of the different roles of the motions of the intracellular and extracellular (particularly in the clefts between the sheets) water on the net observed diffusion effects, would prevent us from confidently applying previously proposed strain-dependent corrections to the diffusion effects that would be expected for a simple homogeneous elastic material [20]. There are also likely differences in the blood content of the muscle in systole and diastole [30], which might also affect the phase-dependent findings. 4) Interpreting the observed diffusion orientation differences as reflecting alterations in the underlying myocyte and sheetlet mobility and associated alterations in contractility or relaxation may not be justified, given all the other uncertainties.

## Discussion/prospects for future CMR DTI studies

While the reported findings of changes in DTI measurements of the apparent sheetlet orientation with cardiac cycle phase, and of differences in this change in HCM patients, are intriguing, more work will need to be done to verify them and to understand the relationship of these findings to the actual underlying tissue microstructure. This will require both changes in the technical imaging methods, e.g. including larger numbers of sampled diffusion-weighted directions in the imaging, and making more direct measurements (e.g. with magnetization tagging) of the associated motion and strain history of the tissue over the cardiac cycle, as well as improvements in the associated analysis methods. Even with better measurements of the regional stain history, there will still be some uncertainty about the effects of the presence of microstructural features, such as the clefts between the sheets, and the distribution of the orientations of such features, on the apparent diffusion of water in the tissue. Extending the imaging methods to enable measurements of diffusion effects that are confined near end-diastole and end-systole would also eliminate much of the uncertainty of the meaning of apparent cardiac cycle phase-dependent changes of whole-cycle diffusion measurements. While the imaging study protocol used was already quite long, thus discouraging any attempt to extend it by including sensitization to diffusion along additional directions, further imaging acceleration may be achievable with methods such as sparsity-based image reconstruction [31]; however, this remains to be implemented and evaluated. Recent advances in the design of gradient systems used for diffusion imaging will also be likely to improve the diffusion measurements [21], but further evaluation is needed. There may also be a potential gain in diffusion imaging information achievable in the same imaging time by replacing some of the averaging of repeated measurements of limited directions of diffusion-sensitized signal, which was used here, by sampling of additional diffusion-sensitized directions, with less averaging [32]. The key issue of the lack of understanding of the relationship between the varying tissue strain over the cardiac cycle and the resulting alterations in the measurements of the apparent diffusion within the tissue remains unresolved, and further work will be needed in this important area. There were no associated histological validation studies performed as part of this study; while not practical for a human study, this information would be an invaluable addition to future animal model studies.

While it has already been well established that DTI measurements can provide distributions of apparent fiber angle with depth in the wall that match those expected from histologic studies, even in vivo, the secondary structure of the heart wall has been harder to characterize, especially in vivo. A compelling aspect of DTI methods is their ability to provide us with information that is immediately related to the microstructure of the heart wall, while other quantitative MR imaging techniques (e.g. T1 and T2 mapping) require more effort (and modeling assumptions) to tie the observations to specific underlying microstructural changes. Extending these DTI methods to the study of dynamic changes in the microstructure of the heart wall in vivo, as the present paper is seeking to do, is likely to lead to greater insight into the roles that these micro-scale structures play in cardiac function, in both health and disease (including HCM), with the hope that they may also provide useful new tools for clinical investigation.
